# The chronic mild stress (CMS) model of depression: History, evaluation and usage

**DOI:** 10.1016/j.ynstr.2016.08.002

**Published:** 2016-08-24

**Authors:** Paul Willner

**Affiliations:** Dept. of Psychology, Swansea University, Swansea, SA2 8PP, UK

**Keywords:** Depression, Chronic mild stress, Validity, Reliability, Neurobiology of stress, Antidepressant, Hippocampus, Prefrontal cortex

## Abstract

Now 30 years old, the chronic mild stress (CMS) model of depression has been used in >1300 published studies, with a year-on-year increase rising to >200 papers in 2015. Data from a survey of users show that while a variety of names are in use (chronic mild/unpredictable/varied stress), these describe essentially the same procedure. This paper provides an update on the validity and reliability of the CMS model, and reviews recent data on the neurobiological basis of CMS effects and the mechanisms of antidepressant action: the volume of this research may be unique in providing a comprehensive account of antidepressant action within a single model. Also discussed is the use of CMS in drug discovery, with particular reference to hippocampal and extra-hippocampal targets. The high translational potential of the CMS model means that the neurobiological mechanisms described may be of particular relevance to human depression and mechanisms of clinical antidepressant action.

## 1 Introduction

Animal models of psychiatric states are procedures applied to laboratory animals which engender behavioural changes that are intended to be homologous to aspects of psychiatric disorders, and can therefore be used as experimental tools to further the understanding of human psychopathology (Willner, 2009). The chronic mild stress (CMS) model of depression (Willner et al., 1992, Willner, 1997) is often considered as a prototypical example. In this model, rats or mice are exposed chronically to a constant bombardment of unpredictable micro-stressors, resulting in the development of a plethora of behavioural changes, including decreased response to rewards, a behavioural correlate of the clinical core symptom of depression, anhedonia. In the canonical version of the model, reward sensitivity is tracked by periodic tests in which the animal is given access to a highly preferred sweet solution, or to a choice between a sweet solution and plain water. Consumption of, or preference for, the sweet reward decreases over weeks of exposure but can be restored to normal levels by chronic treatment with antidepressant drugs.

This paper presents a historical overview of the CMS model, considers some areas of controversy, and reviews recent research in three major areas of application: neurobiological processes mediating the effects of chronic stress, cellular and systemic mechanisms of antidepressant action, and antidepressant drug discovery. The focus of the review is on the specific contributions of CMS research, rather than an integration of CMS studies with the wider literature. The aim, in taking this approach, was to explore how comprehensive a story this single model provides.

## 2 Origins

The origin of the CMS model was in series of studies by Katz and colleagues, published in the early 1980s, in which rats were exposed sequentially to a variety of severe stressors. Most of these studies assessed the effects of stress using changes in open field behaviour, which were reported to be reversed specifically by chronic treatment with antidepressant drugs, but not by non-antidepressants (e.g. Katz and Hersch, 1981, Katz et al., 1981a, Katz et al., 1981b, Katz and Baldrighi, 1982). However, in one study, it was observed that animals exposed to the chronic stress regime failed to increase their fluid consumption when saccharin or sucrose were added to their drinking water, and it was postulated that this might reflect a decrease in the hedonic impact of the sweetener (Katz, 1982). This hypothesis was supported by the demonstration by Anisman and colleagues that uncontrollable footshock can lead to impairments of behaviour maintained by brain stimulation reward (Zacharko et al., 1983, Zacharko et al., 1984). The importance of anhedonia (the decreased ability to experience pleasure) as a core symptom of depression, both then and now (American Psychiatric Association, 2013), stimulated research to develop and validate a model of stress-induced anhedonia as a tool, initially, to study the mechanisms of antidepressant action.

In addition to making hedonic measures the primary focus of the model, a second change to the procedure described by Katz and colleagues was that the severity of the stressors employed was greatly reduced. This change was made for ethical reasons, and in the context of the Animals (Scientific Procedures) Act (1986) that had very recently tightened the framework for animal research in the UK, placing an emphasis on minimizing stress wherever possible. Consequently, the CMS regime as initially developed did not include any of the severely stressful elements used by Katz and colleagues, such as intense footshock, cold water immersion, or prolonged (48 h) food and water deprivation. The birth of the CMS procedure was in a lab meeting in 1986 where those present brainstormed the problems that might arise if animals were curated by a thoroughly incompetent technician: water bottles might leak into the bedding; it might not be noticed that food and water supplies needed replenishment; the light-dark cycle in the animal house, and the lights themselves, might malfunction; housing conditions might change unpredictably; the environment might be noisy; and so on. All of the ideas generated were then implemented in a combination that was unremitting (no stress-free periods) and continuous over many weeks (the chronic element of CMS). For licensing purposes under the 1986 Act, all of the individual micro-stressors were considered to be ‘mild’ but the overall procedure was given a ‘moderately stressful’ banding. The first publication using the CMS procedure reported that rats exposed chronically (5–9 weeks) to CMS, and tested weekly, showed a reduced consumption of, and preference for, weak solutions of saccharin or sucrose, which developed over the course of several weeks exposure, and that the decreased sucrose preference was restored by 2–4 weeks of treatment with a tricyclic antidepressant (Willner et al., 1987).

Over the next few years, this observation was extended to other typical and atypical antidepressants (e.g. Muscat et al., 1992, Cheeta et al., 1994, Papp et al., 1996), and the behavioural effects of CMS were more fully characterized (e.g. Muscat and Willner, 1992, D'Aquila et al., 1994, Cheeta et al., 1997), in particular by the observation that behavioural changes compatible with anhedonia were seen not only in consumption tests but also using place preference conditioning methods (e.g. Papp et al., 1991, Papp et al., 1992), and a comparable CMS procedure was implemented in mice (Monleon et al., 1994). This period also saw the first use of the CMS model to study brain mechanisms underlying antidepressant action, with a particular focus at that time on the mesolimbic dopamine system, which had been somewhat overlooked in earlier depression research (e.g. Stamford et al., 1991, Papp et al., 1994, Dziedzicka-Wasylewska et al., 1997), suggesting a novel direction for antidepressant drug development (e.g. Muscat et al., 1992, Willner et al., 1994). Other labs began to take up the CMS procedure, with a particularly important contribution by Moreau et al., 1992, Moreau et al., 1993 who reported that CMS-induced anhedonia, and its reversal by chronic antidepressant treatment, could also be demonstrated as a change in the threshold for intracranial self-stimulation of the ventral tegmental area. A 1997 review of the first ten years of CMS research identified 12 additional labs that were working with the CMS as applied to rats, half of which had not yet reached the stage of refereed publication, as well as one other lab working with mice (Willner, 1997).

## 3 Uptake

**Fig. 1:**
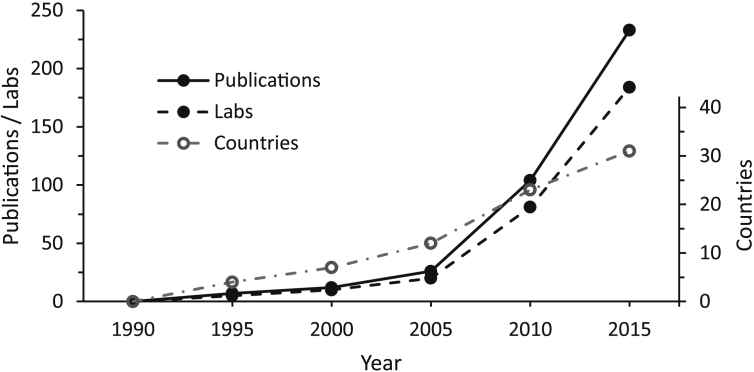
Uptake of the CMS model: 1990–2015.

**Fig. 2:**
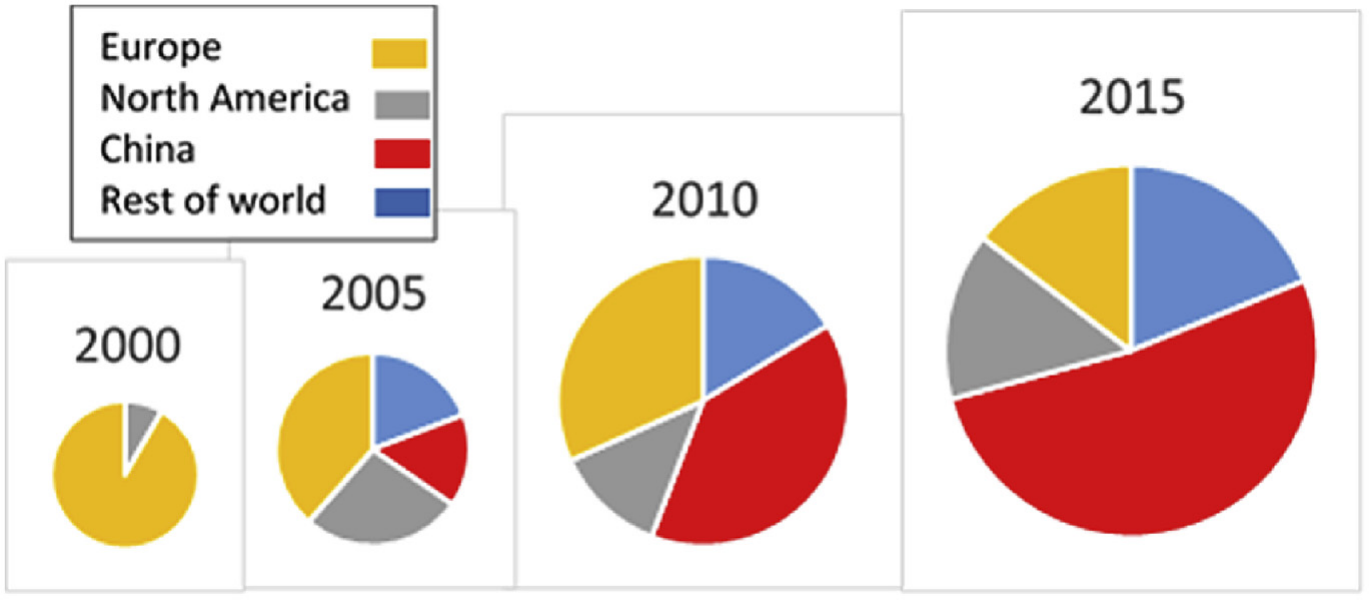
Geographical origin of CMS studies. The size of the circle approximates the volume of publications in each year.

From those beginnings, there has been an exponential growth in the number of laboratories and publications using the CMS procedure, amounting, in 2015 alone, to 230 publications from 180 labs in more than 30 countries (Fig. 1). From the figures shown in Fig. 1, it can be estimated that by the end of 2015 well in excess of 1300 papers using this methodology had been published. The geographical origins of CMS publications are shown in Fig. 2. It can be seen that in addition to the growth in the number of publications, the centre of gravity has shifted from a predominantly European activity in 2000 to a predominance of Chinese publications, amounting to greater than 50% of the total in 2015.

With the exception of two studies in fish, the 230 papers published in 2015 all concerned studies in rats or mice. In order to gain an overview of the scientific focus of CMS studies, these 230 papers were classified, on the basis of the abstract, into three categories: Neuroscience, where the paper included direct measures of brain structure, function or constituents; Other, which comprised studies of stress effects on behaviour or on physiological systems other than the brain, including tests of putative antidepressant agents; and Traditional medicine, which included any publication with a focus on plant-derived or other natural products as well as procedures such as acupuncture, irrespective of the other content of the paper (that is, the Traditional medicine category over-rode the other two categories). Table 1 shows the number and proportion of each type of investigation in different geographical regions. The predominant focus in the western world (Europe and North America) is neuroscientific, while over a third of Chinese investigations are focussed on traditional medicine: however, 78% of the non-Traditional Chinese studies were classified as neuroscience, similar to western studies. This proportion was somewhat smaller in ‘rest of world’ countries, of which Brazil and India were the most prolific (11 and 8 publications from 8 to 7 labs respectively).

## 4 What's in a name?

Although in this paper the model under discussion is referred to generically as CMS, the literature reviewed covers a range of ostensibly different procedures with the common feature that they involve chronic administration of a variety of different stressors. An accompanying paper (Willner, 2017) describes a survey of users of these procedures (as discussed further below). Respondents to the survey variously described their procedure as chronic mild stress (CMS, n = 26), chronic unpredictable stress (CUS or UCS, n = 11), chronic unpredictable mild stress (CUMS or UCMS, n = 25), or chronic variable stress (CVS, n = 9). This raises the question of whether the different names do in fact denote different procedures: specifically, whether the term ‘Unpredictable’ denotes that the procedure is more random, and whether the term ‘Mild’ denotes that the procedure is less severe, than when these terms are not used.

The question of predictability is easily addressed by interrogating the survey responses with respect to whether the micro-stressors making up the stress regime were presented in a fixed order from week to week, or randomly. (It is questionable whether rodents would be able to discern the regularity in a pattern of stressors encountered at weekly intervals, but many labs use random scheduling as a precaution.) The survey received a total of 71 responses, with a roughly equal representation of rat and mouse labs. The majority of labs (75%) employed random presentation, with a marginally greater usage with mice than with rats (85% vs. 66%, p = 0.049). Table 2 summarizes usage of the term ‘Unpredictable’ in relation to random vs. fixed stressor presentation. Overall, a procedure designated as ‘Unpredictable’ is more likely to use random stressor presentation. However, while marginally significant for rats, this relationship is absent for mice because fixed stressor presentation is so rarely used with mice. Thus, the term ‘Unpredictable’ does not add meaning when applied to mouse studies. And while the term is to some extent meaningful when applied to rat studies, almost half of rat studies that were not designated as ‘Unpredictable’ nevertheless used random stressor presentation.

The question of severity is more recondite, because the relative severity of different stress procedures is uncertain in the absence of comparative physiological data. To address this issue, the different micro-stressors included in CMS-type studies were each rated for severity, on a 5-point scale, using an expert consensus procedure described in the accompanying paper (Willner, 2017), and these ratings were used to construct three crude severity indices for each of the chronic stress procedures in the database: variety (the number of micro-stressors applied), severity (the proportion of micro-stressors rated 4 or 5 on the 5-point severity scale), and burden (the total of all ratings). The scores on these three measures are shown in Table 3, separately for rats and mice, for those procedures that were described as ‘Mild’ (CMS, UCMS) and those that were not (CUS, CVS). None of the comparisons differ significantly. As an aside, it has been claimed that the CUS and UCMS procedures differ in that the former has “the advantage of … the absence of stressors that interfere with water and/or food deprivation” (Monteiro et al., 2015). In fact, the opposite appears to be the case: of the 12 labs in the survey that described their procedure as CUS, 10 include water and/or food deprivation in the stress schedule (83%), compared with only 16 of the 25 (64%) that described their procedure as UCMS.

We may conclude from this analysis that the name by which the stress regime is described contains almost no information. With a single modest exception (presentation order in rats) the use, or non-use, of the terms Mild and Unpredictable has no discernible relationship to the content of these stress schedules. The content of the schedules is highly variable, but not in a manner that is reflected in the names by which they are described. Therefore, the differently designated procedures have been treated as equivalent for the purposes of this review.

## 5 Validity

The validity of animal models of psychiatric disorders is usually assessed using a multidimensional approach in which a model is evaluated in respect of its predictive validity (performance in the test predicts performance in the condition being modelled), face validity (there are phenomenological similarities between the two), and construct validity (the model has a sound theoretical rationale). In relation to animal models of depression, predictive validity refers primarily to specific and selective responsiveness to (drug and non-drug) antidepressants; face validity is assessed by comparison to DSM (now DSM-V) symptom checklists, with particular reference to core symptoms of depression; and construct validity is based on an argument for similarity of psychological constructs, such as responsiveness to rewarding events, or if they are known, the underlying neurobiological mechanisms. These principles were first proposed in relation to animal models of depression (Willner, 1984) and the CMS model was the first model to be systematically investigated using this framework, as summarized in a review of the first ten years of CMS research (Willner, 1997).

Regarding its predictive validity, the CMS model was shown in early research to respond to chronic, but not acute, administration of a wide range of established antidepressant drugs (and to electroconvulsive shock), but not to drugs that are clinically ineffective as antidepressants (summarized in Willner, 1997, Table 2). Over the subsequent decades, the pharmaceutical industry has been remarkably unsuccessful in its attempts to bring new and improved antidepressants to the clinic. A few notable exceptions are the very selective SSRI escitalopram, the dual 5-HT/noradrenaline uptake inhibitor venlafaxine, and the 5HT2C/melatonin agonist agomelatine: all of these newer drugs are also effective in the CMS model (Montgomery et al., 2001, Millan et al., 2001, Papp et al., 2003). Also effective are experimental antidepressant strategies that are showing promising results in clinical investigations, including repetitive transcranial magnetic stimulation (e.g. Feng et al., 2012, Wang et al., 2014), deep brain stimulation (e.g. Hamani et al., 2012, Dournes et al., 2013, Lim et al., 2015) and the NMDA glutamate antagonist ketamine (e.g. Garcia et al., 2009, Ma et al., 2013, Papp et al., 2016a).

An attractive feature of the CMS model is the range of behavioural and physiological changes seen following exposure to CMS that parallel symptoms of depression, supporting the face validity of the model. The 1997 review considered the sequelae of CMS in relation to the DSM-IV diagnoses of major depression and melancholia (major depression with melancholic features), diagnoses that are almost unchanged in DSM-V (American Psychiatric Association, 2013). The effects of CMS include, in addition to anhedonia-like impairments in tests of rewarded behaviour, decreases in the performance of other motivated (e.g. sexual and aggressive) behaviours, relative weight loss (i.e., a slower rate of weight gain), disrupted sleep patterns, decreased locomotor activity, and decreased “active waking” in the EEG, all of which parallel symptoms of major depression, as well specific characteristics of melancholia, such as worsening in the early part of the waking phase and a phase advance of circadian rhythms (summarized in Willner, 1997, Table 1). Indeed, it was argued in the 1997 review that “the only symptoms of depression that have not been demonstrated in animals exposed to CMS are those uniquely human symptoms that are only accessible to verbal enquiry” and that by applying the DSM diagnostic rules, “a rat exposed to CMS could, in principle, legitimately attract a DSM-IV diagnosis of either major depressive disorder or major depressive disorder with melancholic features” (Willner, 1997, p.323). Setting aside subjective symptoms such as suicidal ideation that in principle cannot be modelled in animals, there was a single piece missing from the picture: at that time, no parallel had been demonstrated in CMS studies to the clinical symptom of “diminished ability to think or concentrate or indecisiveness”. This gap was subsequently filled by a substantial literature demonstrating cognitive impairments following CMS, in tests as diverse as spatial learning (e.g. Song et al., 2006, Cuadrado-Tejedor et al., 2011) and novel object recognition (e.g. Orsetti et al., 2007, Elizalde et al., 2008). One change from DSM-IV to DSM-V is the recognition in DSM-V that depression may be associated with high levels of anxiety (denoted by the specifier “with anxious distress”). While not universally observed, anxiogenic effects, have frequently been described in animals subjected to CMS (e.g. Surget et al., 2008, Wang et al., 2014, Papp et al., 2016a, Papp et al., 2016b). Like the anhedonic effects, CMS-induced cognitive impairments and anxiogenesis are also reversed by chronic antidepressant treatment.

Broadly speaking, theories of depression have either a psychological or a neurobiological basis (though there are recent attempts to bring these two explanatory frameworks into alignment: e.g. Disner et al., 2011, Willner et al., 2013). Hence there are two routes to construct validity for an animal model of depression. The theoretical rationale for the CMS model from a psychological perspective is that this procedure simulates anhedonia, a loss of responsiveness to pleasant events. This rationale rests on two assumptions, that sucrose drinking is a valid measure of sensitivity to reward, and that CMS causes a generalized decrease in reward sensitivity, rather than a specific effect on responses to sweet tastes. The second of these assumptions is uncontroversial: as mentioned earlier, in addition to a decreased response to sweet tastes, CMS also increases the threshold for brain stimulation reward (e.g. Moreau et al., 1992, Moreau et al., 1993) and impairs the formation of conditioning place preferences to a wide variety of natural or drug reinforcers (e.g. Papp et al., 1991, Papp et al., 1992). The first assumption was challenged by two early reports that decreased sucrose consumption was secondary to a loss of body weight (Matthews et al., 1995, Forbes et al., 1996). However, this critique was comprehensively refuted by many studies demonstrating that decreased sucrose intake or preference could be observed in the absence of body weight loss (including studies that excluded food and water deprivation from the CMS regime), or after taking body weight loss into account (Willner et al., 1996, Willner, 1997; for a recent example: Papp et al., 2016a, Papp et al., 2016b). A further indication that changes in sucrose intake or preference are not secondary to body weight loss is that chronic antidepressant treatment normalizes the hedonic measures but typically does not restore body weight (Willner, 1997). It was also noted that the procedure used in the studies that gave rise to this critique were associated with extreme (>20%) loss of body weight relative to non-stressed controls, and that the results reported may reflect the stress associated with the procedure used to induce weight loss, rather than weight loss per se (Willner, 1997). A more recent point of criticism is that despite their pervasive anhedonia, while depressed patients sometimes report that sweet tastes are less pleasant (e.g. Steiner et al., 1993), more typically they do not (e.g. Dichter et al., 2010). However, “taste” reactivity includes an olfactory component, which is likely to be a much larger factor in rodents, given their greater olfactory sensitivity relative to humans: interestingly, while depressed people may not show taste anhedonia, they do show olfactory anhedonia (Atanasova et al., 2010, Naudin et al., 2012, Naudin et al., 2014). The relative contributions of gustatory and olfactory inputs to the decreased response to sweet tastes in rodents exposed to CMS remains to be established. It may also be relevant to note that a sucrose-drinking session represents a greater source of reward for rodents (relative to other ongoing life experiences) than a human taste test.

The explosion of CMS research (Fig. 2), and the predominance of studies with a primarily neuroscientific focus (Table 1), has resulted in an extensive literature exploring parallels between the physiological mechanisms underlying CMS effects and clinical depression. A systematic review of neurochemical studies published up to 2010 concluded that CMS reliably affects “neurobiological variables that exhibit coincident alterations in clinical populations with major depressive disorder … (including) … upregulation of frontocortical 5-HT2A receptors, downregulation of hippocampal 5-HT1A receptors, upregulation of cortical β-adrenoreceptors, downregulation of hippocampal GR, increases in CRH mRNA in the PVN of the hypothalamus, upregulation of prefrontal cortical cannabinoid CB1 receptors, reductions in frontocortical and hippocampal BDNF protein and reductions in cortical AC-PKA signalling” (Hill et al., 2012, p.2110). There are also morphological parallels between CMS and depression: in particular, the major structural change that is reliably reported in the depressed brain is a decrease in the volume of the hippocampus (Campbell et al., 2004, Videbech and Ravnkilde, 2004), an effect that is also associated with CMS-induced anhedonia (Delgado y Palacios et al., 2011, Luo et al., 2014). As outlined below, studies using the CMS model have made a major contribution to the construction of a theoretical account of psychobiological processes in depression and antidepressant action (Willner et al., 2013), further strengthening the construct validity of the model.

## 6 Reliability

The chronic mild stress (CMS) model of depression is considered by many to be the animal model of depression that has the greatest validity and translational potential, but it has often been criticized for a perceived lack of reliability. Indeed, the view that the procedure is unreliable is widely accepted, and features prominently in reviews of animal models of depression (see Willner, 2017 for examples). However, this position is difficult to reconcile with the fact that the CMS model has been used successfully in, literally, hundreds of laboratories worldwide (see Fig. 1). The study reported in the accompanying paper (Willner, 2017) was undertaken to explore this discrepancy, with the aims of establishing the extent to which the CMS model is reproducible, and identifying experimental variables relevant to its reliability. Because failures to replicate frequently remain unpublished, a survey methodology was used. A questionnaire was circulated to 170 labs identified from a PubMed search as having published a CMS study in the years 2010 or 2015. No selection was applied in respect of the results reported, and recipients were guaranteed anonymity in order to encourage honest reporting of difficulties they may have experienced.

Responses were returned by 71 (42%) of the recipients, followed by further correspondence with some of them. Most of the respondents (n = 53: 75%) reported that the CMS procedure worked reliably in their hands. Of the others, 15 (21%) reported that the procedure was usually reliable, though not always (n = 9: 13%) or not for all measures (n = 6: 8%). Only three respondents (4%) reported being unable to reproduce the characteristic effects of CMS, two of whom may be using an insufficient duration of CMS exposure. The study has the obvious limitation that it was not possible to include laboratories that may have worked with the CMS model but not published their data. However, the overwhelming message from a large sample is that the CMS model does appear to be generally reliable within laboratories and robust across laboratories. Consequently, it appears that the many published statements to the effect that the procedure is unreliable are incorrect.

A series of analyses compared the 75% of ‘reliable’ labs with the 25% of ‘less reliable’ labs on a range of experimenter, subject, stress and outcome variables. Few if any significant differences between these two samples were identified, possibly because of the small size and diversity of the ‘less reliable’ sample. Consequently, the study did not succeed in identifying the critical features of the CMS procedure that result in a less effective implementation in a minority of laboratories. However, from an overview of the survey responses and the published literature, it appears that success with using the model depends on an interplay between (i) individual differences in susceptibility to stress, both within and between animal populations, (ii) the overall severity of the micro-stressors applied, which need to be sufficiently intense to evoke a physiological stress response and sufficiently variable to prevent habituation to their repeated presentation, and (iii) good laboratory practices, which are particularly important when the sucrose test is used as the main outcome measure. The evidence underpinning these conclusions is presented in the accompanying paper (Willner, 2017).

## 7 Individual differences in susceptibility to CMS

Of all the factors that have been studied in relation to the reliability of the CMS model, individual differences have received the greatest attention. Individual differences in susceptibility to CMS have been studied in relation to strain differences, ‘personality’ characteristics, genomic manipulations and natural variation.

The best characterized strain difference is between the more resilient C57BL/6 and more susceptible BALB/c mouse strains (Griffiths et al., 1992, Ducottet and Belzung, 2004, Ducottet and Belzung, 2005, Farley et al., 2012, Palumbo et al., 2010). Increased susceptibility to CMS has also been reported for Flinders Sensitive Line rats relative to the Flinders Resistant Line (Pucilowski et al., 1993), while DBA/2 mice are more even more resilient than C57BL/6 mice (Pothion et al., 2004). This variability in the response to CMS reflects strain differences in anxiety- and depression-relevant behaviours: greater susceptibility to CMS, both between and within strains, has been found to be associated with high levels of anxiety in both rats and mice (Ducottet et al., 2004, Ducottet and Belzung, 2005, Li et al., 2010, Castro et al., 2012), emotionality in rats (Stedenfeld et al., 2011), and submissiveness in mice (Strekalova et al., 2004, Strekalova et al., 2011). There are also anxiety-related differences in susceptibility to CMS among outbred Wistar rats from different suppliers, which could result from genetic drift, different rearing conditions, or a combination of these two factors (Theilmann et al., 2016).

Genomic manipulations have been reported that increase both susceptibility and resilience to CMS. For example, a knockout of the cannabinoid CB1 receptor increased susceptibility to CMS (Valverde and Torrens, 2012), consistent with preclinical and clinical evidence of a protective role of cannabinoid neurotransmission in depression (Gorzalka and Hill, 2011). Conversely, overexpression of the cannabinoid CB2 receptor conferred resilience to CMS (García-Gutiérrez et al., 2010), albeit that pharmacological manipulations of the CB2 receptor failed to alter sucrose intake after CMS in BALB/c mice (Onaivi et al., 2008). Other genomic variants that increase susceptibility to CMS in mice include knockdown of the VGLUT1 receptor, which leads to increased glutamate and decreased GABA levels in the cortex and hippocampus (Garcia-Garcia et al., 2009), and an OCT2 null mutant, which displays an enhanced HPA response to stress (Couroussé et al., 2014). Conversely, several genomic over-expression models present increased resilience to CMS. These include rats that over-express the signal transduction factor ERK2, causing a decrease in the firing of dopamine cells in the ventral tegmental area (Iñiguez et al., 2010), and rats that over-express brain-derived neurotrophic factor (BDNF) in the hippocampus (Taliaz et al., 2011), two systems that are strongly implicated in the behavioural effects of CMS, as described below. Resilience is also shown by mice with over-expression of mineralocorticoid receptors in the hippocampus (Kanatsou et al., 2015), which dampens the HPA response to stress (de Kloet et al., 2016).

There is increasing interest in examining neurochemical differences between subgroups of susceptible and resilient rats (Wiborg, 2013) and mice (Strekalova and Steinbusch, 2010) defined empirically by their behavioural response to CMS. A gene profiling study identified several hundred genes that were differently regulated in mice characterized as vulnerable or resilient to CMS (Strekalova et al., 2011). Similar patterns of gene expression (Christensen et al., 2011) and protein expression (Bisgaard et al., 2007) were observed between animals that were resilient to CMS, and those that developed anhedonia but subsequently responded to antidepressant treatment; these two groups differed in gene and protein expression from animals that developed anhedonia but were treatment-resistant. Proteomic studies have found that susceptible and resilient animals differ, inter alia, in presynaptic proteins involved in neurotransmitter release (Bisgaard et al., 2007, Henningsen et al., 2012, Han et al., 2015) and in levels of neurotrophins, which were decreased in CMS-susceptible animals and increased in resilient animals (Bergström et al., 2008, Farhang et al., 2014). The neurochemical results are not entirely consistent, however: for example, an increased expression of the 5HT transporter has been associated with both susceptibility (Couch et al., 2013) and resilience (Tang et al., 2013) to CMS.

Because CMS-susceptible and CMS-resilient animals are identified post hoc, it can be difficult to determine whether differences between these populations, and between these subgroups and non-stressed animals, represent trait-like individual differences that could play a causal role in the differential behavioural response, or consequences of CMS exposure. For some findings, the neurochemical differences appear consequential to CMS. For example, Zurawek et al. (2013) reported that both stress-susceptible and stress-resilient rats showed a down-regulation of dopamine D2 receptors when exposed to CMS, but with continued exposure D2 receptor density recovered to control levels in resilient animals, while remaining low in susceptible animals. In some other studies, neurochemical parameters that distinguished susceptible and resilient animals were reported to equalize in CMS-susceptible animals that responded to antidepressant treatment (Tang et al., 2013, Li et al., 2014, Nieto-Gonzalez et al., 2015), again, suggesting that the differences reflect consequences of CMS rather than trait-like predisposing factors. Other findings suggest a more trait-like difference. For example, CMS-susceptible animals showed a decrease in enkephalin mRNA expression in the basolateral amygdala, which was not seen in CMS-resilient animals. The trait-like nature of this difference is suggested by the further observation that the effect of CMS was mimicked by a knockdown of enkephalin in the basolateral amygdala (Bérubé et al., 2014). These examples illustrate the difficulty of interpreting studies of empirically defined vulnerable vs. resilient animals, and the need for further investigation to clarify experimental findings.

## 8 Brain mechanisms underlying the effects of CMS

As described above, there has been extensive research on the physiological changes seen in the brains of rats and mice subjected to CMS, and a comprehensive review of this literature described how the effects of CMS on numerous neurochemical systems closely parallel the neurochemical abnormalities described in the brains of depressed patients (Hill et al., 2012). But what initiates these changes? There is a clear answer to this question: perhaps unsurprisingly, the effects of CMS reflect an elevated physiological stress response.

The major stress-responsive system is the hypothalamus-pituitary-adrenal (HPA) axis. The critical role of this system in the effects of CMS is demonstrated by experiments showing that the development of a depressive phenotype during exposure to CMS (decreased sucrose preference and other typical behavioural changes) is blocked by the glucocorticoid receptor antagonist mifepristone (Wu et al., 2007), the corticosterone synthesis inhibitor metyrapone (Kvarta et al., 2015) or adrenalectomy (Goshen et al., 2008, Chen et al., 2016). Conversely, the behavioural and physiological effects of CMS can be mimicked by chronic exogenous administration of corticosterone (Goshen et al., 2008, Gourley and Taylor, 2009, Kvarta et al., 2015), demonstrating that elevated activity of the HPA axis is both necessary and sufficient for the behavioural effects of CMS. While CMS typically increases plasma levels of corticosterone (e.g. Goshen et al., 2008, Kvarta et al., 2015; and numerous other studies) there are also many reports of CMS inducing anhedonic and other depressive-like behaviours in the absence of a detectable elevation of plasma corticosterone (e.g. Willner et al., 1987, Remus et al., 2015; for ancient and modern examples). This probably reflects the fact that corticosterone spikes are elicited in response to the onset of each individual stressor (Sapolsky et al., 1984, Magarinos et al., 1996), making the timing of corticosterone assays critical. The difference between CMS, which induces anhedonia, and repeated administration of the same stressor, which typically does not (e.g. Zhu et al., 2014) is that under repeated presentation of the same stressor, HPA activity habituates, whereas under CMS the acute HPA response to each novel stressor remains intact (e.g. Radley and Sawchenko, 2015).

The systemic mechanisms by which CMS-induced HPA activation results in behavioural impairments are also now reasonably well understood. The critical factor is that HPA activity is held in check by negative feedback systems operating through forebrain structures, with the primary feedback at the level of the hippocampus (Jacobson and Sapolsky, 1991, Herman et al., 1996, Belzung and Billette de Villemeur, 2010). However, chronic exposure to glucocorticoids is neurotoxic, and hippocampal granule cells are particularly sensitive to these effects, leading to a loss of the inhibitory effect of the hippocampus on HPA activity. Effects of CMS exposure on the hippocampus include an initial activation of microglia, a hallmark of neuropathology, which is probably driven by increased exposure to glucocorticoids (Walker et al., 2013): a causative role of this effect is suggested by the fact that minocycline, which blocks microglial activation, also prevents the later development of CMS-induced anhedonia (Kreisel et al., 2014). More prolonged exposure to CMS causes a decreased hippocampal expression of the nuclear transcription factor CREB, leading to decreased expression of BDNF (Grønli et al., 2006, Song et al., 2006) and other neurotrophins (Warner-Schmidt and Duman, 2007, Greene et al., 2009, Elsayed et al., 2012). This loss of trophic support to neuronal structure and function results in shrinkage of the dendritic tree of hippocampal neurons (Sousa et al., 2000, Bessa et al., 2009), and ultimately, loss of granule cells (Jayatissa et al., 2008, Jayatissa et al., 2010). Another relevant factor is that the hippocampus is one of very few brain areas in which neurogenesis continues into adult life, and this process is powerfully suppressed by prolonged exposure to corticosterone or to stressors (Wong and Herbert, 2004, Samuels and Hen, 2011, Petrik et al., 2012), including CMS (Mineur et al., 2007, Oomen et al., 2007). These morphological changes are associated with a decrease in the volume of the hippocampus following CMS (Bessa et al., 2009; Delgado y Palacios et al., 2011), which is the major abnormality reported in structural imaging studies of patients with major depression (Campbell et al., 2004, Videbech and Ravnkilde, 2004). As a consequence of these neurotoxic effects, the outputs of the hippocampus are compromised. The hippocampal inhibitory control of the HPA axis is exerted via multisynaptic pathways projecting from the subiculum to the paraventricular nucleus of the hypothalamus, via the stria terminalis, the lateral septum, and other hypothalamic nuclei (Herman et al., 2005, Belzung and Billette de Villemeur, 2010). In all these structures, changes in neuronal activity secondary to glucocorticoid stimulation of hippocampal neurons were absent in animals subjected to CMS (Surget et al., 2011).

While the hippocampus is the brain area that is most sensitive to the neurotoxic effects of stress, prolonged exposure to stress or high levels of glucocorticoids can also cause damage in many other brain regions, particularly the prefrontal cortex (PFC) (Liu and Aghajanian, 2008, Hinwood et al., 2011, McEwen, 2010, Dias-Ferreira et al., 2009), which, like the hippocampus, also exercises negative feedback over the HPA system (Ulrich-Lai and Herman, 2009, Jankord and Herman, 2008). Consistent with these effects in other stress models, CMS causes atrophy of dendrites on pyramidal cells in the medial PFC (Bessa et al., 2009, Licznerski and Duman, 2013), associated with decreases in prefrontal metabolism (Caldecott-Hazard et al., 1988) and burst firing of pyramidal cells (Guo et al., 2014), and decreased expression of BDNF (Guo et al., 2014, Filho et al., 2015) and synaptic proteins such as PSD95, synapsin I and connexion 43 (Li et al., 2011b, Chen et al., 2016), together with loss of glial cells (Banasr and Duman, 2008, Elsayed et al., 2012) and a decrease in the volume of the PFC (Bessa et al., 2009).

The amygdala is the point of entry through which stress stimulates the HPA axis. In contrast to the negative feedback of the HPA axis that operates primarily through the hippocampus (and, to a lesser extent, the PFC), a positive feedback system operates through the amygdala, such that activation of the amygdala by stress stimulates the HPA axis, which in turn further stimulates the amygdala, the system being held in check by the hippocampus (Myers et al., 2012). Consequently, a loss of hippocampal inhibition over the HPA axis increases activity in the amygdala. CMS has been found to cause an increased coherence of activity in the amygdala (Delgado y Palacios et al., 2014), along with an increase in the length of dendrites and the density of dendritic spines (Li et al., 2015, Sharma and Thakur, 2015, Qiao et al., 2016), and increased expression of CRF (associated with anxiogenesis) and synaptic proteins (Wang et al., 2010, Li et al., 2015). This increased amygdalar activity in turn has consequences for activity in remote brain areas. Of particular interest, activation of the amygdala by CMS causes a decrease in the activity of mesolimbic dopamine cells in the ventral tegmental area (VTA) (Chang and Grace, 2014). The mesolimbic dopamine system has long been of interest in relation to the anhedonic effects of CMS. In particular, CMS has been shown to decrease the release of dopamine in the nucleus accumbens shell in response to reward, while at the same time increasing dopamine release in the same region in response to aversive stimulation (Di Chiara et al., 1999), and to decrease the expression of dopamine D2 receptors in the nucleus accumbens (Papp et al., 1994, Dziedzicka-Wasylewska et al., 1997). Drugs that activate the mesolimbic dopamine system reverse CMS-induced anhedonia (Willner et al., 1994, Papp and Wieronska, 2000), an effect also seen with optogenetic activation of the VTA (Tye et al., 2013). The effect of CMS is transmitted from the amygdala to the VTA via a projection involving the ventral pallidum (Chang and Grace, 2014), which in turn projects to the VTA via the lateral habenula and rostromedial tegmental nucleus (Hikosaka, 2010, Proulx et al., 2014). (A similar amygdala – habenula – VTA pathway has been delineated in the human brain: Ide and Li, 2011.) The lateral habenula is recognized to be a key structure mediating the response to emotionally negative states, via control of the ascending 5HT and mesolimbic dopamine pathways (Hikosaka, 2010, Proulx et al., 2014). Consistent with the effects already described in the amygdala and the mesolimbic system, CMS increases metabolic activity in the habenula (Caldecott-Hazard et al., 1988), and inhibition of this area by deep brain stimulation reverses the behavioural sequelae of CMS (Meng et al., 2011, Lim et al., 2015).

It is evident that CMS influences multiple brain systems, and that the mesolimbic dopamine system, which has been most closely associated with the anhedonic effects of CMS, is affected by CMS via a very indirect route (amygdala – ventral pallidum – lateral habenula – rostromedial tegmental nucleus – VTA – nucleus accumbens). This is perhaps relevant to the observation that decreases in sucrose intake may be more limited (e.g. Henningsen et al., 2009, Strekalova and Steinbusch, 2010) or more difficult to detect (see Willner, 2017) than certain other effects of CMS that may emanate from more proximal structures.

## 9 Mechanisms of antidepressant action in the CMS model

Any analysis of antidepressant action must start with the observation that the canonical action of antidepressants, to increase transmission at monoaminergic synapses by blocking the reuptake of 5HT and NA, is indeed the basis of their clinical action. While many alternative mechanisms have been proposed in recent years, this basic observation was established many years ago by the classic clinical observations of Delgado and colleagues that, following successful antidepressant treatment of depression, the effect in patients treated with specific 5HT reuptake inhibitors (SSRIs) is reversed by a dietary intervention that decreases transmission at 5HT synapses (Delgado et al., 1990, Delgado et al., 1999), while the effect in patients treated with specific NA reuptake inhibitors (NRIs) is blocked by a pharmacological intervention that decreases transmission at catecholaminergic synapses (Delgado et al., 1993, Miller et al., 1996). Similar effects have been reported in the CMS model: chronic treatment with the SSRI citalopram and the NRI desipramine reversed cognitive impairments in animals subjected to CMS, which were in turn blocked by acute antagonism of transmission at 5HT and NA synapses, respectively (Furr et al., 2011, Bondi et al., 2010).

As outlined above, and elsewhere (Hill et al., 2012, Willner et al., 2013, Willner et al., 2014), CMS has a multitude of neurobiological effects, and almost all are reversed by chronic antidepressant treatment. The question, then, is which of those many effects are located within critical pathways from the synapse to behavioural or clinical recovery? As the studies of antagonism of antidepressant effects at 5HT and NA synapses so powerfully demonstrate, this question can be answered by techniques that antagonise specific components of the spectrum of antidepressant effects. This approach was first applied to the CMS model in the context of dopaminergic effects of antidepressant drugs. After chronic treatment, antidepressants of all classes increase the expression and functional sensitivity of D2 DA receptors in the nucleus accumbens (Papp et al., 1994, Dziedzicka-Wasylewska et al., 1997), the terminal integrative area of the mesolimbic DA system (a specific effect that is not seen in other brain regions). The functional significance of this effect was confirmed in a series of studies showing that the recovery from CMS-induced anhedonia following antidepressant treatment was reversed by an acute challenge with low doses of D2 receptor-blocking drugs, which reversed the action of SSRI, NRI or mixed 5HT-NA uptake inhibitors, while having no effect in non-stressed or non-antidepressant-treated animals (Muscat et al., 1990, Muscat et al., 1992, Sampson et al., 1991). As antidepressants do not affect DA uptake (other than a transitory effect in the PFC, where DA is cleared from synapses primarily by the NA transporter: Tanda et al., 1996), the DA-antidepressant interaction probably occurs indirectly: the ascending 5HT and NA projections terminate within the hippocampus, amygdala and PFC, all of which project to the nucleus accumbens shell, where their projections terminate in partially overlapping fields (French and Totterdell, 2002, French and Totterdell, 2003), and on the same cells that also receive a dopaminergic innervation (Sesack and Grace, 2010). Significantly, like the effects of 5HT and NA synthesis blockade described above, the effect of D2 receptor blockade is also seen in patients: as predicted from the CMS studies, administration of a low dose of the D2 antagonist sulpiride caused a profound return of depressed mood in depressed patients successfully treated with SSRIs (Willner et al., 2005).

**Fig. 3:**
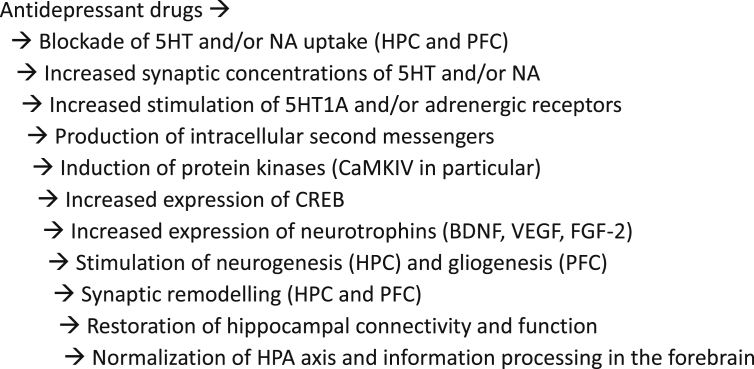
Intracellular and systemic mechanisms of antidepressant action.

The mechanisms by which the primary effect of antidepressant drugs, increased transmission at monoaminergic synapses, gives rise to behavioural changes that may emanate from distant brain areas is now reasonably well understood, and mirrors in large part the picture described above. In brief, antidepressants reverse the neurotoxic effects of stress. As summarized in Fig. 3, and described below, through actions at 5HT1A/5HT2B and noradrenergic receptors, antidepressants influence intracellular second messengers and protein kinases, leading to increased expression of CREB, which in turn increases the expression of BDNF and other neurotrophins that stimulate neurogenesis in the hippocampus and synaptogenesis in the hippocampus and PFC, leading to repair of damaged projections and a rebalancing of information processing in the forebrain. Much of this account can be supplemented by evidence from other animal models (Willner et al., 2013, Willner et al., 2014), but CMS studies alone provide a comprehensive picture. (The CMS model may be unique in this respect.)

A critical role of adult hippocampal neurogenesis was first described in the classic study of Santarelli et al. (2003): following a blockade of cell division by X-irradiation of the mouse hippocampus, the SSRI fluoxetine no longer reversed the behavioural effects of CMS, an observation subsequently extended to the tricyclic antidepressant imipramine (Surget et al., 2008). The same treatment also prevented the restoration of activity in brain areas to which the hippocampus projects, as well as the normalization of the HPA axis (Surget et al., 2011). Through shielding, X-irradiation was confined to the HPC, and blockade of neurogenesis in the other major site of adult neurogenesis, the subventricular zone, was without effect, confirming the crucial role of the HPC (Santarelli et al., 2003). Neurogenesis is under the control of neurotrophins: hippocampal infusions of SU5416, an antagonist of the receptor for the neurotrophin vascular endothelial growth factor (VEGF), also blocked cell proliferation and survival of immature neurons, as well as the restoration of hedonic reactivity, in antidepressant-treated CMS animals (Warner-Schmidt and Duman, 2007, Greene et al., 2009). The significance of neurogenesis in antidepressant action has been criticized (Hanson et al., 2011), a major issue being that suppression of neurogenesis by the toxin methylazoxymethanol acetate (MAM) failed to block the anti-anhedonic effect of a range of antidepressants (Bessa et al., 2009). However, a subsequent study showed that this is a matter of timing: MAM did block antidepressant effects in the CMS model if administered several weeks before the test, which corresponds to the timing used in X-irradiation studies, and suggests that the critical factor is not the genesis of new cells per se, but rather their maturation and incorporation into functional circuits (Mateus-Pinheiro et al., 2013). It appears, therefore, that neurogenesis may be essential for the maintenance of antidepressant effects in the CMS model, but perhaps not for their initiation.

Perhaps more important than neurogenesis for the early stages of recovery from CMS is the effect of synaptogenesis. Antidepressants, both monoamine uptake inhibitors and MAO-A inhibitors, restore the atrophied dendritic tree of hippocampal neurons in animals subjected to CMS (Sousa et al., 2000, Luo and Tan, 2001, Bessa et al., 2009, Licznerski and Duman, 2013, Morais et al., 2014). This effect is associated with a recovery of synaptic function (Kallarackal et al., 2013) and a restoration of hippocampal volume (Bessa et al., 2009, Kong et al., 2009). Neuronal growth is promoted by BDNF and other neurotrophins and behavioural responses to chronic antidepressant treatment (in other experimental models) are blocked in mutant mice with full or forebrain-specific impairment of either BDNF or its receptor, TrkB (Saarelainen et al., 2003, Monteggia et al., 2004), or by a region-specific knockdown of BDNF in the dentate gyrus or ventral subiculum of the hippocampus (but not in the CA1 or CA3 fields) (Adachi et al., 2008, Taliaz et al., 2010). Consistent with these observations in other models, behavioural recovery in the CMS model was also blocked by knockdown of BDNF (Ibarguen-Vargas et al., 2009) or by a TrkB inhibitor (Li et al., 2011a, Yi et al., 2014, Mao et al., 2014, Luo et al., 2015).

Working back towards the synapse, the expression of BDNF, VEGF, and other neurotrophins, is controlled by the nuclear transcription factor CREB. The expression of CREB in the hippocampus is increased by chronic, but not acute administration of antidepressants of all classes (Nibuya et al., 1995, Duman and Monteggia, 2006, Martinowich et al., 2007). In an important CMS study by Kong et al. (2009), the ability of chronic fluoxetine treatment to reinstate CREB phosphorylation was blocked in mice with a knockout of the water channel protein AQP4, which also failed to show other effects of chronic fluoxetine downstream from CREB, including stimulation of neurogenesis and reversal of anhedonic and other behavioural effects of CMS. This study also clarified the upstream pathway from synaptic receptors to CREB. CREB activity is under the control of several second messenger systems, including phosphokinase A and C (PKA, PKC), extracellular signal-related kinases (ERK1/2) and calcium/calmodulin-dependent protein kinase IV (CaMKIV). In the AQP4 knockout animals, CMS and fluoxetine had no effect on PKC activity; PKA and ERK1/2 activities were decreased by CMS and reinstated by fluoxetine; but fluoxetine failed to reverse CMS-induced inhibition of CaMKIV activity, suggesting that this system provides the critical pathway from the synapse to the nucleus.

With the exception of neurogenesis, which is specific to the hippocampus, a similar story is developing in the PFC. For example, CMS has been shown to decrease glial cell proliferation in the PFC, which was reinstated by chronic antidepressant treatment (Banasr et al., 2007). The expression of fibroblast growth factor-2 (FGF-2) is also decreased by CMS in the PFC specifically, and the effects of antidepressant treatment to reinstate sucrose consumption and gliogenesis were blocked by local administration of a FGF receptor antagonist (Elsayed et al., 2012) or by a toxic ablation of PFC astrocytes (Banasr and Duman, 2008). Antidepressants also remediate the dendritic atrophy and spine loss of PFC pyramidal cells in CMS animals (Bessa et al., 2009; Mateus Pinheiro et al., 2013; Licznerski and Duman, 2013), with recovery of lost PFC volume (Bessa et al., 2009) and a restoration of burst firing by pyramidal neurons, which was suppressed by CMS (Guo et al., 2014). These effects are associated with – and presumably, driven by – changes in PFC BDNF expression, which is decreased by CMS and restored by chronic antidepressant treatment (Guo et al., 2014, Filho et al., 2015, Jin et al., 2015, Tang et al., 2015b).

The importance of the PFC as a target for antidepressant action is highlighted by the effectiveness of deep brain stimulation of the ventromedial PFC in treatment resistant depression (Mayberg, 2009, Hamani et al., 2011), together with the significance of the PFC as the site of antidepressant action of the NMDA receptor antagonist ketamine, which is claimed to effect rapid improvements in antidepressant refractory patients following a single intravenous infusion (Berman et al., 2000, Zarate et al., 2006, Diazgranados et al., 2010). In the CMS model, a single injection of ketamine caused a rapid reversal, within 2 h, of the anhedonic and other behavioural effects of CMS. Within 24 h, a single ketamine injection also reversed the loss of synaptic proteins and atrophy of dendritic spines in the PFC and the associated electrophysiological deficits (Li et al., 2010, Li et al., 2011b, Duman et al., 2012, Tang et al., 2015a). These effects appear to be mediated by the rapid stimulation by ketamine of the mammalian target of rapamycin (mTOR), because they were blocked, in the CMS model, by the selective mTOR inhibitor rapamycin (Li et al., 2010, Li et al., 2011b, Duman et al., 2012). The mTOR pathway is localized in neuronal dendrites and spines and contributes to activity-dependent synaptic plasticity via the synthesis of proteins for new synapse formation (Hoeffer and Klann, 2010). The precise mechanism by which ketamine interacts with mTOR to reverse CMS effects is unclear because ketamine does not alter mTOR activity (Li et al., 2010, Li et al., 2011b, Tang et al., 2015a), but it does decrease the activity of the downstream effectors of mTOR (Tang et al., 2015a).

The ketamine story has heightened interest in glutamatergic synapses in the PFC as a potential extra-hippocampal and non-monoaminergic target for antidepressant action. Antidepressant-like effects of NMDA antagonists were described in early CMS studies (Klimek and Papp, 1994, Papp and Moryl, 1994), and more recent CMS studies have reported rapid antidepressant-like effects, comparable to those of ketamine, for selective antagonists at the NMDA-1B subunit (Li et al., 2011b) and the glycine site on the NMDA receptor (Zhu et al., 2013), as well as a partial agonist at the glycine site (Burgdorf et al., 2015). Glial cells in the PFC have an important role in removing glutamate from the extracellular space, and as noted above, antidepressant action in the CMS model was blocked by ablation of astrocytes in the PFC (Banasr and Duman, 2008). Consistently, an antidepressant-like effect was seen with riluzole, which decreases activity at glutamatergic synapses by decreasing presynaptic release of glutamate and facilitating uptake by glial cells (Banasr et al., 2010). In contrast to these reports, antidepressant-like effects in the CMS model have also been reported for an inhibitor of the glycine transporter GlyT1 (Depoortère et al., 2005), and following epigenetic induction of mGlu2 receptors (Nasca et al., 2013), two treatments that might be expected to potentiate glutamatergic transmission at NMDA receptors (Cubelos et al., 2005, Tyszkiewicz et al., 2004). These discrepancies await resolution.

## 10 CMS and drug discovery

The CMS model was originally developed as a platform for investigating neurobiological mechanisms, as discussed in the two preceding sections, rather than as a drug discovery vehicle, for which it is ill-suited by virtue of the long duration and labour-intensive nature of CMS experiments. Nevertheless, use of the CMS model has been widely adopted within drug discovery and development programmes. Analysis of publications retrieved in the PubMed search (Fig. 1, Fig. 2) suggests that overall around 20% of CMS publications report studies of potential novel antidepressants, with an increase since 2010 to 30% in 2015, reflecting the growth in studies involving traditional Chinese medicines (Table 1). From these figures it can be estimated that there are more than 300 publications describing the use of the CMS model in a drug development context. This total excludes studies of conventional antidepressants, ketamine and brain stimulation, as well as the many unpublished studies conducted by or on behalf of drug companies. It is beyond the scope of the present paper to review in detail this extensive literature, but two general trends are apparent.

The major driver of the search for novel antidepressants has been the failure over many years to achieve significant improvements in efficacy, delay of onset, or proportion of depressed patients responding to treatment, prompting a search for drugs acting other than by potentiating transmission at monoaminergic synapses. The growing evidence that antidepressants act by repairing a damaged hippocampus provided an alternative starting point, leading to many new approaches aimed at achieving the same end by different means. These include drugs that act as HPA antagonists (to decrease the threat to the hippocampus by dampening the response to stress), neuroprotective agents (to protect the hippocampus against glucocorticoid-induced neurotoxicity), or promoters of neurogenesis and synaptogenesis (to repair the damage). Table 4 lists some HPA-antagonist and neuroprotective agents for which antidepressant effects have been reported in both CMS and clinical studies. (It is important to add that some of the clinical effects listed are far from well established: the table is intended simply to illustrate the variety of approaches.) The CMS literature also includes a panoply of further antioxidant and other neuroprotective agents that have not been tested clinically. However, while many drugs have been identified that have antidepressant effects and beneficial effects on the hippocampus, there is minimal evidence that any of them improve on conventional antidepressants in respect of their efficacy, onset of action, or, for the drugs that have been tested clinically, their effectiveness in treatment-resistant patients. And this is hardly surprising: conventional antidepressants provide an effective and efficient repair service and there is no theoretical or empirical basis for predicting that their competitors would do the job better (Willner et al., 2014).

As discussed earlier in this review, and elsewhere (Willner et al., 2013, Willner et al., 2014), stress-induced hippocampal damage leads to changes in the functioning of distant brain regions that are more directly involved in depressive psychopathology. Accordingly, an alternative strategy is to consider targets outside the hippocampus. Table 5 lists interventions for which antidepressant efficacy has been reported, in both CMS experiments and clinical trials, through actions at extra-hippocampal sites (and not directly involving potentiation of NA or 5HT transmission). For each intervention, the table lists a single clinical reference (for all except the final three entries there is an extensive clinical literature) as well as studies reporting antidepressant-like actions in the CMS model. The interventions include the use of deep brain stimulation to inhibit activity in not only the ventromedial prefrontal cortex, but also the lateral habenula, ventral tegmental area and medial forebrain bundle, as well as activation of the dorsolateral prefrontal cortex by repeated transcranial magnetic stimulation (rTMS). The melatonin MT1/MT2 agonist and 5-HT2C antagonist agomelatine is one of very few agents with greater efficacy than other antidepressants: the site of action of agomelatine is unknown, but the lateral habenula is a strong candidate (Willner et al., 2013). Antidepressant effects are also reported for drugs that potentiate activity in the mesolimbic dopamine system, through presynaptic or postsynaptic actions (atypical antipsychotics and D2/3 agonists, respectively). The final entries in the table are drugs targeting the glutamate system, which, as discussed above, include, in addition to ketamine, the glutamate release inhibitor riluzole, the mGlu2 enhancer l-acetylcarnitine, and the glycine receptor partial agonist rapastinel. In some cases the CMS experiments were conducted to corroborate clinical observations, while in others, the CMS studies were part of the developmental trajectory preceding the clinical trials.

Of particular significance, most of the interventions listed in Table 5 are claimed to act more rapidly than conventional antidepressants, and with a single exception (agomelatine), all of them are claimed to be effective in treatment-resistant depression. (The clinical studies cited in Table 5 were chosen to illustrate this point.) Indeed, for ethical reasons, most of these interventions (agomelatine, the dopaminergic drugs and rTMS are exceptions), have only been tested in patients who failed to respond to antidepressant treatment. In common with other animal models of depression, the CMS model can report that a novel intervention is antidepressant-like, but, because the model responds to conventional antidepressants, it cannot predict whether a novel intervention will also be effective in patients who are treatment-resistant. Consequently, while CMS may be an excellent model for studying problems relevant to depressive features that can be treated with conventional antidepressants, it is less than ideal as a tool for investigating the specific features of treatment-resistant depression. A solution to this problem may be to implement the model in treatment-resistant animals. This strategy is discussed in detail elsewhere (Willner and Belzung, 2015): its feasibility has been demonstrated (Dournes et al., 2013), and further studies along these lines are to be expected.

One final point worth making is that the chronic time-course of CMS experiments makes it possible to distinguish between anxiolytic and antidepressant drugs. Anxiolytics act by dampening activation of the amygdala (Forster et al., 2012), and consequently, anxiolytic drugs prevent CMS effects when administered from the onset of CMS (e.g. Zhao et al., 2012). However, unlike antidepressants, anxiolytics fail to reverse established CMS effects when drug treatment commences after several weeks of CMS and CMS remains in operation alongside drug treatment (e.g. Muscat et al., 1992). Consequently, antidepressant activity cannot be inferred from experiments in which drug treatment and CMS start concurrently. While this procedure can decrease the duration of a CMS experiment by several weeks, it should not be used in studies of the potential antidepressant activity of novel compounds.

## 11 Conclusions

After a slightly rocky start, the CMS model has become firmly established as an indispensable experimental tool for studying the neurobiological basis of depression. Recent research has added further to the validity of the model, and early concerns about its reliability appear to have largely receded. The model has made substantial contributions to our understanding of the neurobiological consequences of chronic stress and the reversal and repair of those effects by chronic antidepressant treatment. It is also evident that the CMS model has become established as a valuable component of antidepressant drug discovery and development programmes. Of course, there is much more to say about these problems: the aim of this review was identify some specific contributions of CMS research, not to present a comprehensive account of the neurobiology of depression and antidepressant action (see Hill et al., 2012, Willner et al., 2013, Willner et al., 2014), and certainly not to dismiss other experimental approaches. But for those issues that have been addressed, the high translational potential of the CMS model provides grounds for optimism that the positions outlined here, with respect to neurobiological mechanisms underlying the chronic effects of stress and antidepressants in rodents, also describe processes of relevance to human depression and mechanisms of clinical antidepressant action.

## Figures and Tables

**Table 1:** Distribution of CMS studies in 2015.

	Number in 2015^a^				Proportions		
	N	O	T	Total	%N	%O	%T
Europe	26	8	0	34	76	24	0
North America	24	10	0	34	71	29	0
China	60	16	42	118	51	14	36
Rest of world	20	22	2	44	45	50	5

a Publications are coded as: involving traditional medicine products or procedures, including studies of their neurobiological basis (T); other studies with a neurobiological focus (N); and other non-neurobiological studies: for example, use in drug discovery (O).

**Table 2:** Proportion of labs using random stressor presentation.

Unpredictable?	Overall	Mice	Rats
No (CMS/CVS)	61%	87%	48%
Yes (CUMS/UCMS/UCS)	87%	92%	86%
	p = 0.009		p = 0.036

p = Fisher exact probability test.

**Table 3:** Estimates of stress severity in relation to whether a stress procedure is described as “mild” a.

	Mice		Rats	
Mild?	Yes	No	Yes	No
n	24	6	23	12
Variety	7.08 (0.35)	7.50 (0.76)	7.41 (0.38)	7.25 (0.46)
Severity	40.4 (3.5)	39.3 (5.3)	26.5 (2.5)	38.0 (5.4)
Burden	21.0 (1.2)	21.8 (2.6)	20.1 (1.1)	21.8 (1.5)

a Values are mean (standard error) For this analysis, six response were excluded where respondents reported using both mice and rats, because it was uncertain whether the stress regime reported was applied to one species or to both.

**Table 4:** Antidepressant activity via neuroprotection a.

	CMS	Depression
**HPA inhibitors**		
CS synthesis inhibition	Kvarta et al., 2015	Kling et al., 2009
CS receptor antagonism	Wu et al., 2007	Gallagher et al., 2008
CRF antagonism	Ducottet et al., 2003	Zobel et al., 2000
Cytokine antagonism	Goshen et al., 2008	Raison et al., 2013
Oestrogen	Romano-Torres and Fernández-Guasti, 2010	Keating et al., 2011
**Anti-oxidants**		
Omega-3 pufas	Vancassel et al., 2008	Sarris et al., 2016
Ascorbic acid	Moretti et al., 2012	Amr et al., 2013
S-adenosylmethionine	Benelli et al., 1999	Sarris et al., 2016
Melatonin	Haridas et al., 2013	Fava et al., 2012
Curcumin	Liu et al., 2014	Sanmukhani et al., 2014

a For each target the table lists a single example from each of the CMS and clinical literatures.

**Table 5:** Extra-hippocampal sites of antidepressant action a.

Brain site	Treatment	CMS	Depression
Lateral habenula	Deep brain stimulation	Lim et al., 2015	Sartorius et al., 2010
	Agomelatine?	Papp et al., 2003, Boulle et al., 2014, Rossetti et al., 2016	Taylor et al., 2014
Ventral tegmental area	DA D2 antagonists	Papp and Wieronska, 2000	Kriston et al., 2014
Nucleus accumbens	DA D2 agonists	Willner et al., 1994	Lattanzi et al., 2002
	Deep brain stimulation	Gersner et al., 2010	Bewernick et al., 2012
Medial forebrain bundle	Deep brain stimulation	Furlanetti et al., 2015	Galvez et al., 2015
dl-Prefrontal cortex	Repeated transcranial magnetic stimulation	Feng et al., 2012, Wang et al., 2014, Kim et al., 2014	Rossini et al., 2005
vm-Prefrontal cortex	Deep brain stimulation	Hamani et al., 2012, Lim et al., 2015, Dournes et al., 2013	Mayberg, 2009
	Ketamine	Li et al., 2010, and many other papers	Coyle and Laws, 2015
	Riluzole	Banasr et al., 2010	Zarate et al., 2004
	L-acetylcarnitine	Nasca et al., 2013	Bersani et al., 2013
	Rapastinel	Burgdorf et al., 2015	Preskorn et al., 2015

a For each target, the table lists CMS studies and provides a single example from the clinical literature.
